# Development and Evaluation of Epitope-Blocking ELISA for Detection of Antibodies against Contagious Caprine Pleuropneumonia in Goat Sera

**DOI:** 10.3390/vetsci6040082

**Published:** 2019-10-18

**Authors:** Baziki Jean de Dieu, Bodjo S. Charles, Nick Nwankpa, Ethel Chitsungo, Cisse Rahamatou Moustapha Boukary, Naomi Maina, Takele A. Tefera, Rume Veronica Nwankpa, Nduta Mwangi, Yao Mathurin Koffi

**Affiliations:** 1 African Union-Pan African Veterinary Vaccine Centre (AU-PANVAC), P.O. Box 1746, Debrezeit, Ethiopia; nickN@africa-union.org (N.N.); ethelC@africa-union.org (E.C.); boukaryc@africa-union.org (C.R.M.B.); 2 Molecular Biology and Biotechnology Department, Pan African University Institute for Basic Sciences, Technology and Innovation (PAUSTI), JKUAT Main Campus, P.O. Box 62000-00200, Nairobi, Kenya; nmaina@jkuat.ac.ke; 3 Biochemistry Department, Jomo Kenyatta University of Agriculture and Technology (JKUAT), P.O. Box 62000-00200, Nairobi, Kenya; 4 Research and Development Department, National Veterinary Institute, P.O. Box 19, Debrezeit, Ethiopia; takeletefera99@gmail.com; 5 Department of Microbial Cellular and Molecular Biology, Addis Ababa University, P.O. Box 1176, Ethiopia; verarume2000@yahoo.com; 6 Foot and Mouth Disease Department, Kenya Veterinary Vaccines Production Institute (KEVEVAPI), P.O. Box 53260-00200, Nairobi, Kenya; mwangi_nduta@yahoo.com; 7 Laboratoire Central Veterinaire, Laboratoire de Virologie, B.P. 206 Bingerville, Côte d’Ivoire; dougbe_yao@yahoo.fr

**Keywords:** blocking-ELISA, cut-off value, monoclonal antibody, sensitivity and specificity

## Abstract

Enzyme linked immunosorbent assays (ELISAs) have been developed for the detection of antibodies against contagious caprine pleuropneumonia (CCPP), the causative agent of which is *Mycoplasma capricolum* subsp. *Capripneumoniae* (Mccp). The currently available commercial CCPP competitive ELISA (CCPP cELISA) kit produced and supplied by IDEXX Company (Westbrook, Maine, United States) is relatively expensive for most African laboratories. To address this issue and provide a variety of choices, a sensitive and specific blocking-ELISA (b-ELISA) test to detect antibodies against CCPP was developed. We describe the newly developed CCPP blocking-ELISA based on the blocking of an epitope of a monoclonal antibody (Mccp-25) by a positive serum sample against the Mccp protein coated on a plate. The Percentage Inhibition (PI) cut-off value for the CCPP b-ELISA was set at 50 using 466 CCPP negative and 84 CCPP positive small ruminant sera. Of the negative sera, 307 were obtained from the Botswana National Veterinary Laboratory (BNVL) and 159 from the Friedrich-Loeffler-Institute (FLI) Germany. The 84 positive sera samples came from experimentally vaccinated goats at the AU-PANVAC facility in Debre-Zeit, Ethiopia. The relative diagnostic sensitivity and specificity of the CCPP b-ELISA was 93% and 88%, respectively. This test result indicated good correlation with that of the commercial CCPP cELISA by IDEXX Company (Westbrook, Maine, United States) with a Cohen’s κ agreement of κ agreement of 0.85. The newly developed CCPP b-ELISA will be useful in the detection of antibodies for the diagnosis CCPP and for sero-surveillance during vaccination campaigns.

## 1. Introduction

Contagious caprine pleuropneumonia (CCPP) is a devastating disease of domestic goats and some wild ungulate species which poses major risks for goat farming in parts of Africa, the Middle East, and Asia [1,2]. Mortality and morbidity rates can reach as high as 60%–80% and 80%–100%, respectively, especially when the disease first enters a territory through carrier animals [3,4]. The disease is caused by the *Mycoplasma capricolum* subsp. *Capripneumoniae* (Mccp) [5], which was first isolated in Kenya [6]; and subsequently in Chad, Eritrea, Ethiopia, Niger, Oman, Sudan, Tanzania, Tunisia, Turkey, Uganda, the United Arab Emirates, and Mauritius [7].

Clinically, CCPP affects the respiratory tract and is characterized in its acute form by fever, anorexia, and severe respiratory distress with coughing, nasal discharge, dyspnea, polypnea, and fibrinous pleuropneumonia with straw-colored pleural fluid [8,9]. The clinical diagnosis of CCPP needs to be differentiated from other diseases affecting small ruminants with similar symptoms, such as pasteurellosis and Peste des Petits Ruminants (PPR). Laboratory diagnosis for the confirmation of CCPP outbreaks is based on culture, isolation, and characterization of Mccp as well as serological tests such as indirect hemagglutination (IHA) [10] and enzyme linked immunosorbent assay (ELISA) [11] for the detection of antibodies. However, most African veterinary laboratories regularly face difficulties in purchasing laboratory reagents and test kits such as ELISAs due to either financial constraints or difficulty in accessibility. In order to address this issue by providing affordable and accessible ELISA tests, a sensitive and specific ELISA test for the detection of CCPP antibodies was developed in this study. This paper describes the development and evaluation process of the CCPP blocking- ELISA (CCPP b-ELISA), which can be used as an alternative assay for sero-surveillance and the detection of antibodies against CCPP in goats.

## 2. Material and Methods

### 2.1. Ethical Approval

All applicable international, national, and institutional guidelines for the care and use of animals were strictly adhered to. AU-PANVAC is a specialized technical agency of the African Union Commission with a host country agreement with the Government of the Federal Democratic Republic of Ethiopia. Consequently, all laboratory activities were conducted in accordance with the laws and regulations of Ethiopia. Animal manipulations were conducted under FDRE (2017): Federal Democratic Republic of Ethiopia, National animal welfare strategy and implementation plan (2017–2022), and the AU-PANVAC Quality Management System.

### 2.2. Preparation of Mycoplasma Capricolum Subsp. Capripneumoniae (Mccp) Antigen

The *Mycoplasma capricolum* subspecies *capripneumoniae* (Mccp) antigen was produced from the Mccp F-38 vaccine strain [12] from the AU-PANVAC (Debrezeit, Ethiopia) vaccine seed repository as per the protocol previously described [13] with slight modifications. Briefly, the CCPP vaccine seed was reconstituted in 5 mL of pleuropneumonia-like organisms (PPLO) broth (Difco, Sparks, MD, USA) without serum and filtered through a 0.45-µm syringe filter. The flow-through was inoculated in PPLO media supplemented with 20% heat inactivated horse serum (GIBCO, Waltham, MA, USA) and 10% yeast extract (Difco, Sparks, MD, USA). The inoculated media was incubated at 37 °C for 10–14 days without shaking and with continuous pH monitoring. When the pH reached between 6.65 and 6.90, the culture was again inoculated into fresh PPLO medium at a ratio of 1/10 culture to fresh PPLO medium and incubated at 37 °C for up to 10 days until the desired turbidity and pH were observed. The CCPP antigen was prepared by centrifuging the final culture at 10,000× *g* for 30 min at 4 °C. The supernatant was discarded and the pellet washed three times with sterile phosphate buffered saline (PBS) (Sigma-Aldrich, St. Louis, MO, USA) by centrifuging at the same speed for 30 min to remove non-specific proteins from the culture media. The pellet was re-suspended in an adequate volume of sterile PBS (approximately 30 mL) and lysed with 0.1% Triton-X (Sigma-Aldrich, St. Louis, MO, USA) overnight at 4 °C. The antigen was finally titrated by chessboard titration as previously described [14,15,16,17] with the Mccp25-horse radish peroxidase (HRP) conjugate (AU-PANVAC, Debre-Zeit, Ethiopia) to determine the optimal dilutions of both the antigen and conjugate for use in setting up CCPP b-ELISA.

### 2.3. Serum Samples

A total of 466 negative sera samples provided by the Botswana National Veterinary Laboratory (BVI), Gaborone, Botswana (307) and the Friedrich-Loeffler-Institute (FLI), Greifswald-Insel Riems, Gemany (159), respectively, were used for the assay development. Meanwhile 252 sera samples obtained from experimentally vaccinated goats at AU-PANVAC were also tested.

### 2.4. Monoclonal Antibody Production, Its Conjugation, and Evaluation in Epitope Blocking

In a recently completed study [18], monoclonal antibodies (mAbs) against Mccp in the CCPP vaccine were generated and characterized at AU-PANVAC Laboratories (Debre-Zeit, Ethiopia). One of these mAbs (Mccp25) was used to develop the CCPP blocking-ELISA. This mAb was predominantly expressed and isotyped to belong to IgG2a, a subtype of immunoglobulin G. This Mccp-25 mAb was conjugated with HRP (Horse Redish Peroxodase) using the established method described in the manufacturer’s instructions for the EZ-Link Plus Activated Peroxidase kit l (PIERCE Cat. 31487; Thermo Scientific, Rockford, IL, USA). The conjugated mAb (Mccp25-HRP) was tested with CCPP b-ELISA and was easily blocked by antibodies from serum samples from CCPP vaccinated goats. The optimal dilution of the Mccp25-HRP conjugate and of the serum samples used in the CCPP b-ELISA was determined by testing two-fold serial dilutions of known strong and negative serum samples.

### 2.5. CCPP Blocking-ELISA Development and Procedure

Greiner Microlon 600 High binding polystyrene ELISA plates (Greiner Bio-One GmbH) were used to setup the CCPP Blocking ELISA (CCPP b-ELISA). The plates were coated overnight at room temperature (21–25 °C) with 100 μL of the Mccp antigen at a dilution of 1:100 in phosphate buffered saline (PBS). The following day, plates were washed three times with 300 μL wash buffer (0.002 M PBS containing 0.05% Tween 20 (PBS-T) to remove the unbound antigen followed by blocking with 200 μL blocking solution (PBS-T containing 5% skimmed milk) for 30 min in a shaker incubator at 37 °C. The blocking solution was poured off and washed 3 times prior to adding 80 μL of the blocking solution followed by 20 μL of the control and serum samples into their respective wells according to the plate layout. The plates were then incubated for 1 h at 37 °C followed by the washes as indicated. In total, 100 μL of Mccp25-HRP conjugate at 1:100 dilutions in blocking buffer was added to each well prior to incubation for 45 min at 37 °C. Following 3 washes as above, 50 μL/well of 3, 3′, 5, 5′-tetramethylbenzidine (TMB) (Thermo Scientific, Rockford, IL, USA) was added and the reaction mixture incubated at 37 °C for 15 min in the dark for color development. The reaction was stopped with 100 µL of sulfuric acid (1 M) and the (Optical Density) *ODs* were read using a spectrophometer with a 450 nm filter. Control buffer (*CB*), positive control (PC), and negative control (*NC*) were included in the test. ODs in wells were converted into percentage inhibition (PI) using the formula below with median *OD* of *NC* (*OD_NC_*) and median *OD* of *CB* (*OD_CB_*).

$$\mathrm{PI}(\%)=100-\left(\frac{{OD}_{Sample}-{OD}_{CB}}{{OD}_{NC}-{OD}_{CB}}\right)\times 100$$

### 2.6. Evaluation of CCPP Blocking ELISA

#### 2.6.1. Analytical Sensitivity

The smallest amount of the antibody that can be accurately detected by the CCPP b-ELISA was defined by testing two-fold serial dilutions starting from a 1:2.5 dilution of strong CCPP-positive and PPR-negative serum samples [19].

#### 2.6.2. Cut-Off Value Determination

The cut-off value was defined by testing 466 negative small ruminant sera from CCPP free countries (307 from Botswana and 159 from Germany). In addition to these were 84 CCPP positive sera from experimentally vaccinated goats obtained at AU-PANVAC. The experimental sera were also tested with the commercial CCPP cELISA kit [20].

#### 2.6.3. Assay Repeatability

Preliminary evidence of repeatability (agreement between replicates within and between runs of the assay) was necessary to warrant further development of the assay [21,22]. This was accomplished by evaluating the results from replicates of all samples in each plate (intra-plate variation), and inter-plate variation using the same sample runs in different plates within a run and between runs of the assay [23]. To determine repeatability, two positive sera samples were tested in 12 replicates per each sample per plate in two runs. The percentage coefficient of variation (CV%) of the optical density data results was determined as previously described [24,25,26].

#### 2.6.4. Determination of Diagnostic Sensitivity and Specificity

To compare and correlate the results between the newly developed CCPP b-ELISA and the available commercial CCPP cELISA, a total of 252 experimental sera (negative and positive) were tested by both tests and the results compared (Table 1).

The relative diagnostic sensitivity and specificity of CCPP b-ELISA were also determined using the experimental sera.

The test results were analyzed and compared to determine the relative sensitivity and specificity of CCPP b-ELISA. Cohen’s κ statistic previously described [27], was calculated to determine the agreement between the results of the two diagnostic tests.

## 3. Results

### 3.1. Titration of Mccp Antigen and Conjugate Used in the CCPP b-ELISA

**Figure 1:**
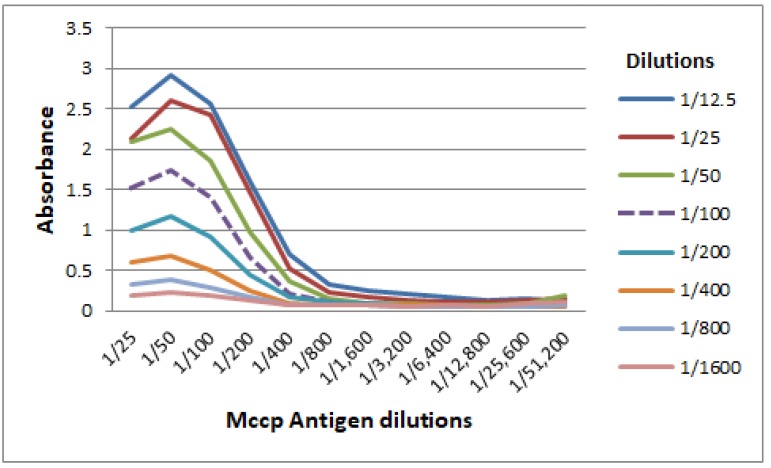
Chessboard titrations of the *Mycoplasma capricolum* subsp. *Capripneumoniae* (Mccp) antigen against the Mccp25-horse readish peroxidase (HRP) conjugate. The optical dilution chosen for both coating the antigen and conjugate was 1:100. This was to ensure that the optical density value (absorbance) ranged from 0.5 to 2.

The chessboard titration of CCPP b-ELISA antigen prepared was performed using the Mccp25-HRP conjugate. An optimal dilution of 1:100 for both antigen and conjugate was determined for use in the CCPP b-ELISA (Figure 1).

### 3.2. Cut-Off Value Determination

**Figure 2:**
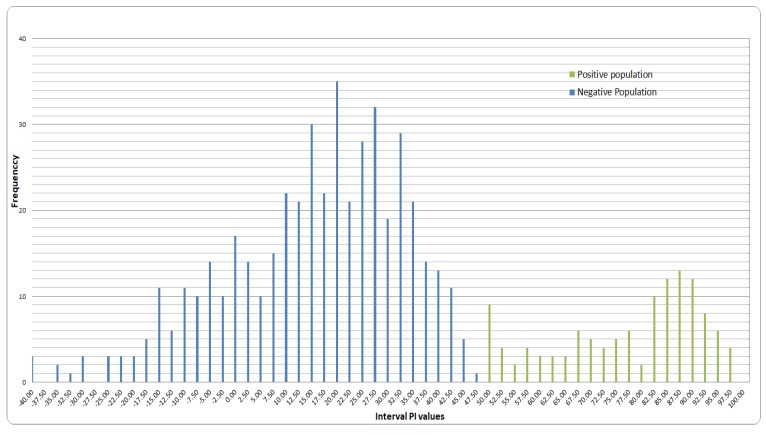
Distribution of Percentage Inhibition (PI) values obtained by CCPP b-ELISA for 466 negative sera samples from the negative populations (blue plots) and 84 positive sera samples from the experimentally vaccinated goats (green plots). Median value for PIs from negative population was 14.21% with standard deviations (SD) at 17.7 47. The highest PI value from the negative sera was 47.5% which was close to the cut-off value established at median value + 2 × SD (14.21 + 2 × 17.7 = 49.80 PI).

The Gaussian distribution (also known as normal distribution) of PIs (blue plots) from the 466 sera tested by the new CCPP b-ELISA is presented in Figure 2. The highest PI value in the negative population was 47.5% PI. The median value of PIs was determined at 14.21% with standard deviation (SD) at 17.7. The cut-off value was established at 50% using the median value + 2 × SD (14.21 + 2 × 17.7 = 49.80 PI). The PI values for the 84 positive serum samples tested with the CCPP b-ELISA were all above the cut-off value of 50% showing clear separation between negative and positive population (Figure 2).

### 3.3. Analytical Sensitivity and Blocking Epitope by Serum Samples

**Figure 3:**
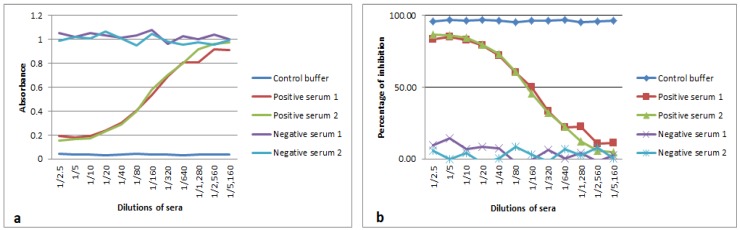
The smallest amount of specific antibodies against contagious caprine pleuropneumonia (CCPP) detected by CCPP blocking-ELISA (b-ELISA) in the sera samples tested. (**a**) The limit of detection for the new CCPP b-ELISA showed the dilution 1:160 for positive sera 1 and 2 (lines red and green). (**b**) The cut-off value for PI at 50 % as the limit of the detection for these two samples.

Using the cut-off value for PI at 50%, the highest dilution of positive sera detected by the new CCPP b-ELISA was established at 1:160 (Figure 3). No blocking reaction was observed with dilutions of negative sera samples used in the assay.

### 3.4. Assay Repeatability

The assay repeatability was determined by testing 12 replicates of the positive sera samples using a single and separate operator for each day. The same was daily and the overall percentage of the coefficient of variation of 5.2% was obtained.

### 3.5. Comparison of the Newly Developed CCPP b-ELISA with the Available Commercial CCPP cELISA

In order to evaluate the diagnostic performance, the CCPP b-ELISA was compared with the cELISA test using a total of 252 goat sera samples (Table 2).

### 3.6. Diagnostic Sensitivity and Specificity

To evaluate the diagnostic reliability of the newly developed CCPP b-ELISA, it was compared with the CCPP cELISA using 252 positive goat sera samples (Table 3).

## 4. Discussion

The CCPP b-ELISA was designed and developed as an alternative test for the detection of antibodies against CCPP in goat sera samples. This test is simple, quick, and generates reliable results. In the present development, a coating antigen was prepared and optimized, and used to produce the Mccp monoclonal antibody (Mccp-25) against the Mccp protein; an immunogenic part of the CCPP [21,22] was also produced. This mAb was conjugated with the HRP enzyme, which removed one step from the other routine ELISA steps making it quicker.

The diagnostic performance of the CCPP b-ELISA in terms of its sensitivity and specificity was also evaluated in comparison with CCPP cELISA which is the gold standard. Using the two-way contingency table for the 252 sera samples tested, 84 were positive and 142 were negative in both tests. Overall, for the 252 samples tested, a sensitivity of 93% was obtained using the b-ELISA out of which 6 sera samples tested negative for the CCPP cELISA while 20 sera samples tested negative with the CCPP b-ELISA, where the specificity was estimated to be 88%. The total false positive and false negative rates indicated by the newly developed b-ELISA were 2.35% and 7.9%, respectively. There were differences in the results for 26 of the samples between the two assays (Table 3). Using the CCPP cELISA as a gold standard, the CCPP b-ELISA results for 20 of the 104 positive samples could be erroneous. In contrast, 6 of the 162 CCPP cELISA negative samples could be due to the different methods used for antibody detection between the two assays. The calculated Cohen k coefficient value of 0.85 indicated a good correlation between the results of the two tests. The repeatability determined from this new method through the determination of the percentage of the coefficient of variation (CV%) was 5.2%, which was within the acceptable range for the repeatability of a diagnostic test [18]. Therefore, these results confirm that the newly developed CCPP b-ELISA can effectively detect CCPP antibodies in goat serum samples. The b-ELISA method developed is simple, rapid, and can be easily accessed from Ethiopia by African laboratories. Ethiopia is a major hop for the African aviation industry with a daily reach to almost all African countries.

## 5. Conclusions

In a nutshell, a blocking ELISA for detection of antibodies against CCPP disease was developed and is based on the blocking of an epitope of a monoclonal antibody (Mccp-25) by a positive serum sample against the Mccp protein coated on plate. The newly developed CCPP b-ELISA diagnostic performances are 93% of sensitivity and 88% of specificity. Its comparison with the current commercial competitive CCPP ELISA indicated a good correlation between the results of the two tests. Further laboratory testing of Mccp-25 mAb for cross-reactivity with other mycoplasma species should be conducted.

## Figures and Tables

**Table 1:** Calculation of diagnostic sensitivity and specificity.

Type of Test	Tested (+)	Tested (−)
Test (+)	T+	F−
Test (−)	F+	T−
	$Sensitivity\;\left(se\right)=\left(\frac{T+}{\left(T+\right)+\left(F-\right)}\right)*$	$Specificity\;\left(Sp\right)=\left(\frac{T-}{\left(T-\right)+\left(F+\right)}\right)$

T+: True positive, T−: True negative; F−: false negative; F+: false positive.

**Table 2:** Comparative test results for the commercial CCPP competitive ELISA (cELISA) and CCPP b-ELISA. The PI cut-off values for the CCPP cELISA and CCPP b-ELISA were 55 and 50, respectively.

Sera	Commercial CCPP cELISA		CCPP b-ELISA	
	Positive ≥ 55 PI	Negative < 55 PI	Positive ≥ 50 PI	Negative < 50 PI
252	90	162	104	148

**Table 3:** A two-way contingency table representing the results obtained for each assay for the 252 sera samples tested. This table enabled the determination of the diagnostic sensitivity and specificity for the CCPP b-ELISA considering CCPP cELISA as the gold standard. The diagnostic sensitivity and specificity for the CCPP-ELISA was 93% and 88% respectively. The Cohen’s κ statistic value was 0.85, which indicated good agreement between the results of the two assays.

Newly Developed Test	Commercial CCPP cELISA			Total
		Positive	Negative	
**CCPP b-ELISA**	Positive	84	20	104
	Negative	6	142	148
	Total	90	162	252
